# An immune-related lncRNA pairs signature to identify the prognosis and predict the immune landscape of laryngeal squamous cell carcinoma

**DOI:** 10.1186/s12885-022-09524-1

**Published:** 2022-05-14

**Authors:** Lvsheng Qian, Tingting Ni, Bing Fei, Hui Sun, Haosheng Ni

**Affiliations:** 1 Department of Otolaryngology, Affiliated Hospital of Nantong University, Nantong, 226001 Jiangsu China; 2 Department of Oncology, Nantong Tumor Hospital, Nantong, 226001 Jiangsu China; 3 Department of Otorhinolaryngology Head and Neck Surgery, Affiliated Huai’an Hospital of Xuzhou Medical University, Huai’an, 223002 Jiangsu China; 4 Department of Pathology, Affiliated Hospital of Nantong University, Nantong, 226001 Jiangsu China; 5 Department of Otolaryngology, Affiliated Hospital of Nantong University, Nantong, 226001 Jiangsu China

**Keywords:** Laryngeal squamous cell carcinoma (LSCC), The Cancer Genome Atlas (TCGA), Long non-coding RNA (lncRNA), Tumor-infiltrating immune cell, Prognostic signature

## Abstract

**Background:**

Laryngeal squamous cell carcinoma (LSCC) is the most common squamous cell carcinoma. Though significant effort has been focused on molecular pathogenesis, development, and recurrence of LSCC, little is known about its relationship with the immune-related long non-coding RNA (lncRNA) pairs.

**Methods:**

After obtaining the transcriptome profiling data sets and the corresponding clinical characteristics of LSCC patients and normal samples from The Cancer Genome Atlas (TCGA) database, a series of bioinformatic analysis was conducted to select the differently expressed immune-related lncRNAs and build a signature of immune-related lncRNA pairs. Then, the effectiveness of the signature was validated.

**Results:**

A total of 111 LSCC patients and 12 normal samples’ transcriptome profiling data sets were retrieved from TCGA. 301 differently expressed immune-related lncRNAs were identified and 35,225 lncRNA pairs were established. After univariate Cox analysis, LASSO regression and multivariate Cox analysis, 7 lncRNA pairs were eventually selected to construct a signature. The riskscore was computed using the following formula: Riskscore = 0.95 × (AL133330.1|AC132872.3) + (-1.23)  × (LINC01094|LINC02154) + 0.65 × (LINC02575|AC122685.1) + (-1.15)  × (MIR9-3HG|LINC01748) + 1.45 × (AC092687.3|SNHG12) + (-0.87)  × (AC090204.1|AL158166.1) + 0.64 × (LINC01063|Z82243.1). Patients were classified into the high-risk group (> 1.366) and the low-risk group (< 1.366) according to the cutoff value (1.366), which is based on the 5-year riskscore ROC curve. The survival analysis showed that the low-risk group had a better prognosis (*P* < 0.001). The riskscore was better than other clinical characteristics in prognostic prediction and the area under the curves (AUCs) for the 1-, 3-, and 5-year survivals were 0.796, 0.946, and 0.895, respectively. Combining age, gender, grade, stage, and riskscore, a nomograph was developed to predict survival probability in LSCC patients. Then, the riskscore was confirmed to be related with the content of tumor-infiltration immune cells and the model could serve as a potential predictor for chemosensitivity.

**Conclusion:**

We successfully established a more stable signature of 7 immune-related lncRNA pairs, which has demonstrated a better prognostic ability for LSCC patients and may assist clinicians to precisely prescribe chemo drugs.

**Supplementary Information:**

The online version contains supplementary material available at 10.1186/s12885-022-09524-1.

## Introduction

Laryngeal squamous cell carcinoma (LSCC) is the most common squamous cell carcinoma of the head and neck and the second most prevalent malignant tumor of the respiratory tract [1]. Important risk factors for LSCC include smoking, drinking, low body mass index, laryngopharyngeal reflux, etc. [2]. LSCC treatment mainly integrates surgery, radiation, and chemotherapy [3]. However, the survival rates for most patients with advanced LSCC have not improved significantly in the last 30 years [4]. The larynx plays a central role in maintaining physiologic processes, including respiration, speech [5]. Therefore, understanding the molecular mechanisms underlying the occurrence and development of LSCC is essential for its diagnosis, treatment, and prognosis.

Long non-coding RNAs (lncRNAs) are a class of RNA molecules that are longer than 200 nucleotides in length and rarely encode for proteins [6, 7]. Recently, the accumulation of lncRNAs has been implicated in physiological and pathological processes, especially in cancers [8]. Specifically, lncRNA SATB2-AS1was reported to inhibit colorectal cancer (CRC) cell metastasis and regulate the immune response of CRC by cis-activating SATB2 [6]. LncRNAs have also been shown to promote malignant biological behaviors [3] and chemotherapy resistance in LSCC [1, 9]. Growing evidence suggests that lncRNAs may be potential biomarkers and therapeutic targets in various cancer types [10]. However, to the best of our knowledge, no prognostic signature of immune-related lncRNA pairs has been studied in LSCC.

Herein, we constructed a signature of immune-related lncRNA pairs and validated its effectiveness in LSCC.

## Materials and methods

### Data acquisition and preprocessing

Transcriptome profiling data sets and the corresponding clinical characteristics of LSCC patients and normal samples were obtained from The Cancer Genome Atlas (TCGA) database (https://portal.gdc.cancer.gov).

### Differential expression analysis and construction of lncRNA pairs

Immune-related genes were screened according to the immune-related gene list obtained from the IMMPORT data source (https://www.immport.org/resources). Immune-related lncRNAs were selected by co-expression analysis based on the expression levels of lncRNAs and immune-related genes in the downloaded samples (|correlation coefficient|> 0.4, *P* < 0.001). Differently expressed immune-related lncRNAs were identified based on the previously selected expression level of lncRNAs between tumor and non-tumor specimens (|logFC|> 1.5, FDR < 0.05). The identified differently expressed immune-related lncRNAs were used to develop differential immune-related lncRNA pairs. The differential immune-related lncRNAs were cyclically singly paired. In the sample, when the level of expression of the first lncRNA was higher compared to the second, it was marked as 1, otherwise, it was marked as 0. Matches were considered effective when the expression level of 0 or 1 lncRNA pairs was between 20 and 80% of the total pairs.

### Cell cultures and quantitative real-time PCR

Quantitative real-time PCR was carried out to verify the expression of these genes. The human HEK293T and LSCC cell line TU686 were purchased from BeNa Culture Collection (BNCC, Beijing, China). TU686 cells were cultured in RPMI 1640 medium (Procell Life Science &Technology, Wuhan, China) supplemented with 10% fetal bovine serum (FBS) (Procell) . HEK293T cells were cultured in DMEM (Procell) containing 10% fetal bovine serum, 100 U/mL penicillin, and 100 μg/mL streptomycin. All cells were cultured at 37˚C under 5% CO2. Total RNA was isolated from cell lines using TRIzol reagent (Invitrogen, Shanghai, China). The cDNA was synthesized using 2×NovoScript Plus 1 st Strand cDNA Synthesis SuperMix (Novoprotein, Suzhou,China). The 20 μL reaction system contained 10 μL SYBR (Vazyme Biotech, Nanjing, China), 0.5μL forward primer, 0.5 μL reverse primer, 1.0 μL cDNA, and 8.0 μL DEPC water. The amplification program was as follows: initial denaturation step at 95◦C for 2 mins, followed by 45 cycles at 95◦C for 10 s, and 60◦C for 45 s. The primers were designed and synthesized by(Sangon Biotech , Shanghai, China). The expression of each gene was normalized by expression of GAPDH. We selected 2 lncRNA pairs from 7 lncRNA pairs.The primer sequences used in this study were as follows: GAPDH forward: 5’ -ATGGCCTTCCGTGTCCC-3’; GAPDH reverse: 5’‐GAGGAGTGGGTGTCGCTGT‐3’. MIR9-3HG forward: 5’-GCTGGGCTTCTCCTGCAATTC-3’; MIR9-3HG reverse: 5’-AACCCTGTAAACCCCTCCTCTCAC-3’. LINC01748 forward: 5’-GCACTCCACAGAAGGAAGGTTCAG-3’; LINC01748 reverse: 5’-GGAAGTGATTGCCAGGAACAGGAC-3’; AC092687.3 forward: 5’-TGGAGTCCGTGCTCAGGTATGG-3’, ACO92687.3 reverse: 5’- ACGCTTGGTGGATGACTTCTTATGC-3’; SNHG12 forward: 5’-CTGAGGAGGTGAGCTTGTTTCTGG-3’, SNHG12 reverse: 5’-GTCCTTGCCTTCTGCTTCCCATAG-3’.

### Construction of the prognostic signature and calculation of the risk score

Based on clinical data of LSCC patients in the TCGA, univariate Cox analysis was used to screen lncRNA pairs related to prognosis. LASSO regression was then performed to further select valuable lncRNA pairs. Then, multivariate Cox regression was performed to construct a prognostic signature of lncRNA pairs and calculate the coefficients. The riskscore of this signature was computed using the following formula: Riskscore = ∑ coef (pair_i_) × value (pair_i_) (i = 1-n), where coef indicates the contribution to the risk score and value represented the lncRNA pair results,0 or 1, in the sample. The 1-, 3-, and 5-year receiver operating characteristic (ROC) curves of the model were plotted.

### Validation of the established risk model

The cutoff value was calculated based on the 5-year survival ROC curve and used to stratify patients into high- and low-risk groups. The survival state was compared between the two groups. Univariate and multivariate Cox regression analyses were performed to confirm whether the riskscore could independently predict the prognosis of LSCC patients. The prognostic relationship between the riskscore and other clinical parameters was explored. A nomograph was developed for predicting survival probability in LSCC patients.

### Correlation analysis of tumor-infiltrating immune cells and riskscore values

The infiltration estimation results for all TCGA tumors were downloaded from the TIMER2.0 database (http://timer.cistrome.org/). These results were analyzed with TIMER [11], CIBERSORT, CIBERSORT-ABS [12], QUANTISEQ [13], xCell [14], MCP-counter [15], and EPIC [16] methods. Spearman correlation analysis was employed to analyze the relationship between tumor-infiltrating immune cells and riskscore values. Meanwhile, the differences in the expression level of immune-related genes were compared between the high- and low-risk groups.

### Analysis of the significance of the model in the clinical treatment

To explore the clinical value of the model, the half-maximal inhibitory concentration (IC_50_) of common chemotherapeutic drugs, such as cisplatin, bleomycin, and paclitaxel, in the TCGA LSCC dataset was calculated. The Wilcoxon signed-rank test was used to analyze the difference in the IC_50_ between the high- and low-risk groups, using the pRRophetic package in R.

### Statistical analysis

LncRNA expression in the tumor and normal tissues was compared by the Wilcoxon test. Univariate and multivariate Cox proportional hazard regression analyses were utilized to explore the correlation between riskscore values and overall survival (OS). The Kaplan–Meier method was used to evaluate the OS. A log-rank test was used to compare the differences between the two groups. Results from at least three independent experiments were presented as mean ± standard deviation (SD). Student’s t test was used to determine the significance of differences in groups.The statistical analysis software included R 4.0.3 and Graphpad Prism 6.0. A *P* value < 0.05 was considered significant.

## Results

### Data acquisition and preprocessing

**Fig. 1:**
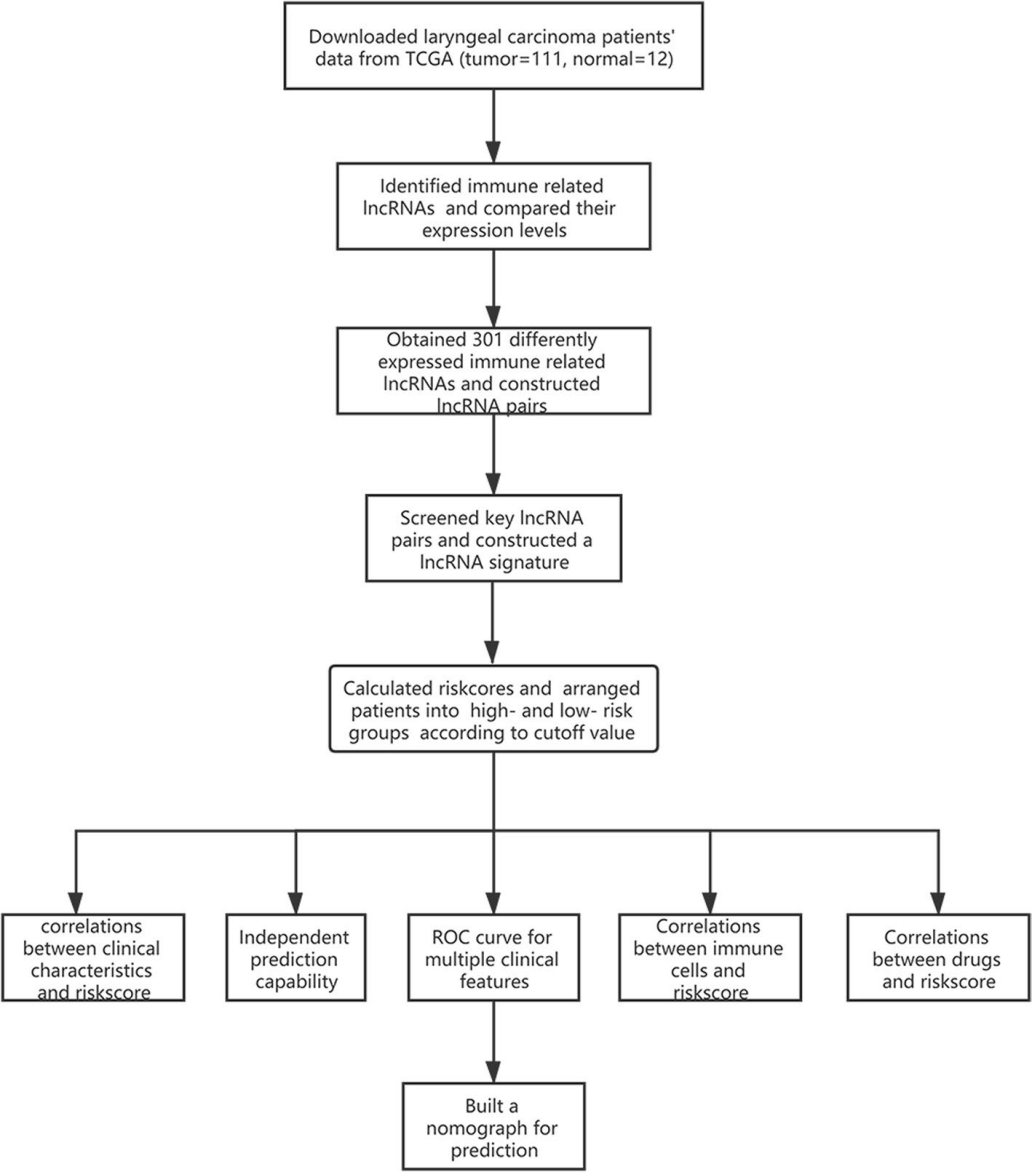
Flow chart of the study

LSCC transcriptome profiling data sets comprising of 111 patients and 12 normal samples were retrieved from TCGA(Table1) . Patients’ age ranged from 38-83 years. Ninety patients were males and 21 were females. Fifty patients were deceased. The flowchart of the study is shown in Figure 1.

**Table 1:** Clinicopathological features of 111 LSCC patients

Characteristics	Cases
**ID**	
< = 65	73(65.8)
> 65	38(34.2)
**Age**	
FEMALE	20(18.0)
MALE	91(82.0)
**Gender**	
G1	8(7.2)
G2	70(63.1)
G3	29(26.1)
GX	4(3.6)
**Grade**	
I	2(1.8)
II	9(8.1)
III	14(12.6)
IV	71(64.0)
unknow	15(13.5)
**T**	
T1	7(6.3)
T2	12(10.8)
T3	25(22.5)
T4	54(48.6)
TX	13(11.7)
**M**	
M0	40(36.0)
M1	1(0.9)
MX	70(63.1)
**N**	
N0	39(35.1)
N1	12(10.8)
N2	39(35.1)
N3	2(1.8)
NX	19(17.1)
**Survival**	
yes	61(55.0)
no	50(45.0)

### Identification of the immune-related lncRNAs and construction of lncRNA pairs

**Fig. 2:**
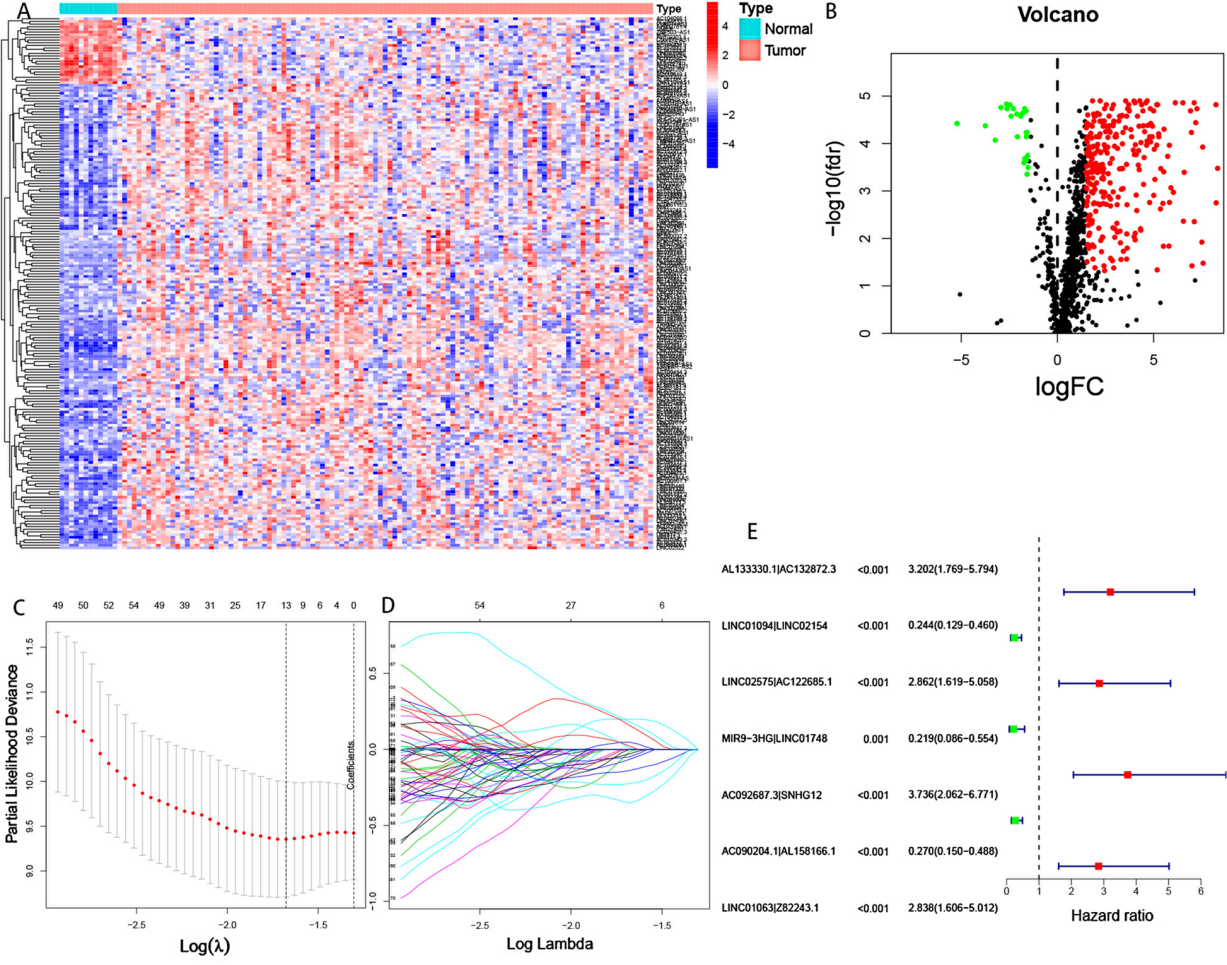
Identification of differently expressed immune-related lncRNAs and construction of a lncRNA pairs signature. **A** The heatmap and (B) volcano plot of the differently expressed immune-related lncRNAs. **C** The generalized cross-validation curve of paired likelihood deviance. **D** The generalized cross-validation curve of coefficients. **E** A forest map showed 7 differently expressed immune-related lncRNA pairs identified by Cox proportional hazard regression in the stepwise method

A total of 1,710 immune-related mRNAs (Supplementary File 1) were selected according to the gene list downloaded from the IMMPORT database, followed by co-expression analysis of known immune-related genes and lncRNAs. Overall, 1105 immune-related lncRNAs (Supplementary File 2) and 301differently expressed immune-related lncRNAs (Supplementary File 3) were identified (Figure 2A, B). Using iterative cycling and 0-or-1 matrix screening, 35,225 lncRNA pairs were established (Supplementary File 4).

### Construction of the prognostic signature of immune-related lncRNA pairs

**Fig. 3:**
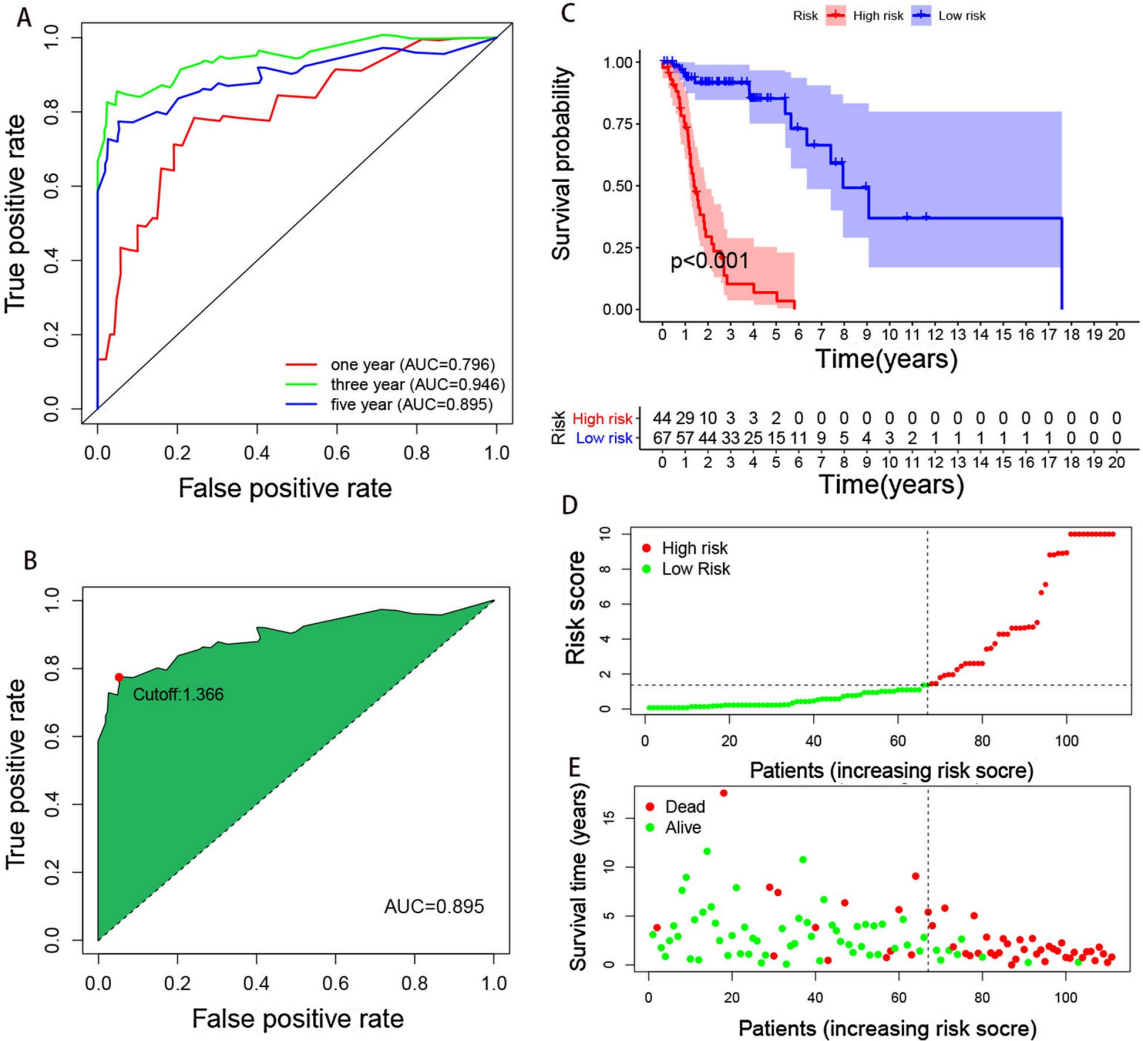
The cut-off value and survival curve between high- and low-risk groups. **A** The 1-, 3-, and 5-year ROC of the optimal model. **B** The maximum inflection point is the cut-off point obtained by the AIC in 5-year ROC curve. **C** The survival curve between high- and low-risk groups. **D** Riskscores and survival outcome (E) of each case

After univariate Cox analysis and 711 prognosis-related lncRNA pairs were identified. Further screening by modified LASSO regression retrieved 13 lncRNA pairs (Fig. 2C, D). Multivariate Cox analysis was then performed and 7 lncRNA pairs were eventually selected to construct a signature of immune-related lncRNA pairs (Table 2). The riskscore was computed using the following formula: Riskscore = 0.95 × (AL133330.1|AC132872.3) + (-1.23) × (LINC01094|LINC02154) + 0.65 × (LINC02575|AC122685.1) + (-1.15) × (MIR9-3HG|LINC01748) + 1.45 × (AC092687.3|SNHG12) + (-0.87) × (AC090204.1|AL158166.1) + 0.64 × (LINC01063|Z82243.1). A forest map of the 7 lncRNA pairs is shown in Fig. 2E. Each sample was scored using this formula. The ROC curves for 1-, 3-, and 5- years were drawn using the riskscore values (Fig. 3A). To verify the expression of these lncRNAs in LSCC, we selected two pairs of more common lncRNA pairs for qRT-PCR, and the results were shown in Supplementary Fig. 1A,B, which showed that the expression of these genes was elevated in LSCC and in accordance with our matching rules.

**Table 2:** Seven prognostic immune-related lncRNA pairs identified from univariate Cox regression analysis, LASSO regression, and multivariate Cox regression analysis

lncRNA pair	Coefficient	HR	95% CI lower	95% CI higher	P value
AL133330.1|AC132872.3	0.95	2.58	1.32	5.02	0.01
LINC01094|LINC02154	-1.23	0.29	0.14	0.61	0.00
LINC02575|AC122685.1	0.65	1.91	1.03	3.54	0.04
MIR9-3HG|LINC01748	-1.15	0.32	0.12	0.85	0.02
AC092687.3|SNHG12	1.45	4.25	2.16	8.35	0.00
AC090204.1|AL158166.1	-0.87	0.42	0.22	0.79	0.01
LINC01063|Z82243.1	0.64	1.90	1.01	3.58	0.05

### Validation of the established risk model

**Fig. 4:**
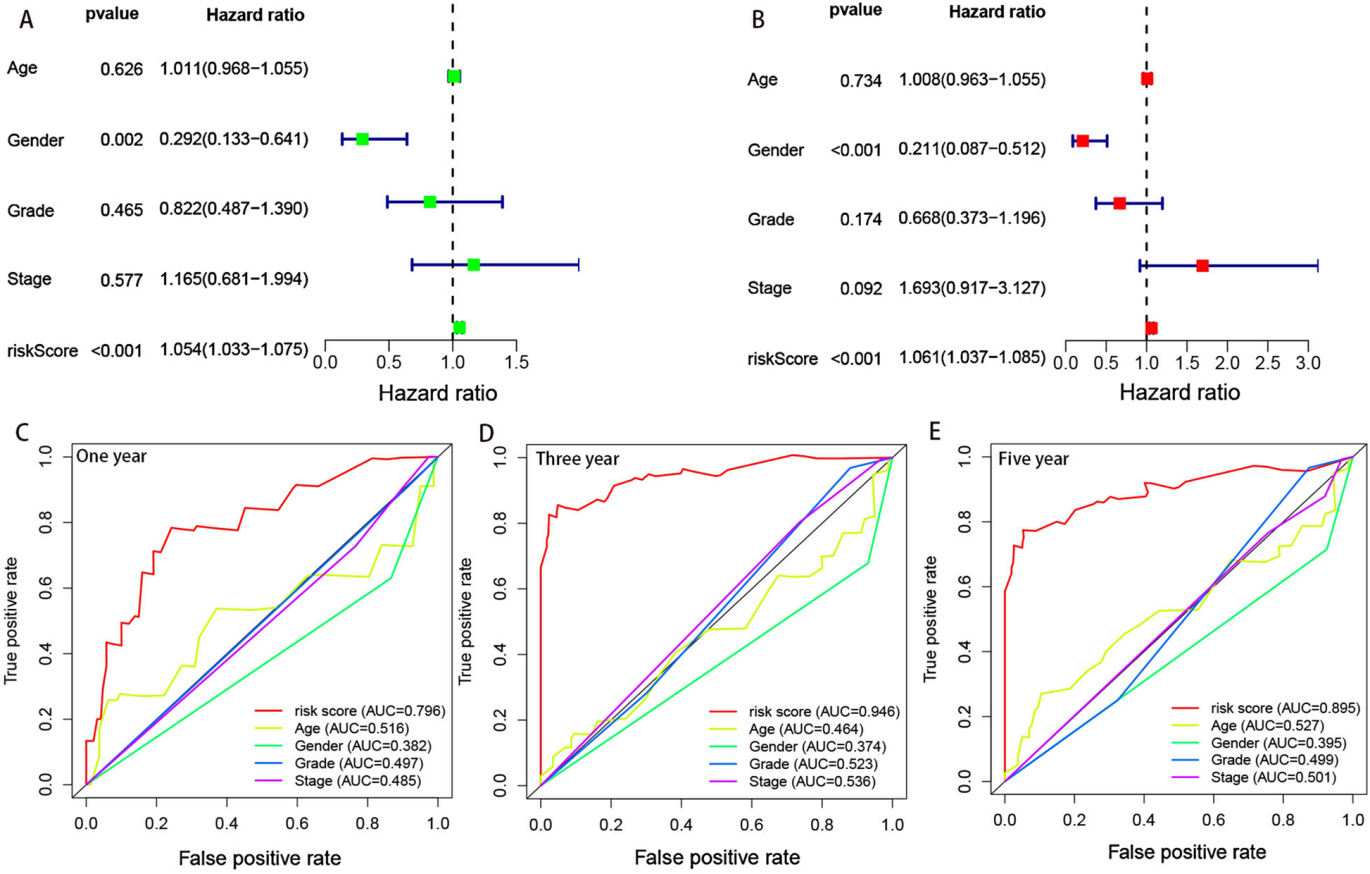
Riskscore was an independent factor in predicting prognosis. (A)Univariate and (B) multivariate Cox regression analysis results were showed by forest maps. (C-E) Riskscore was better than other clinical characteristics in prognostic prediction in 1,3,5- year.

**Fig. 5:**
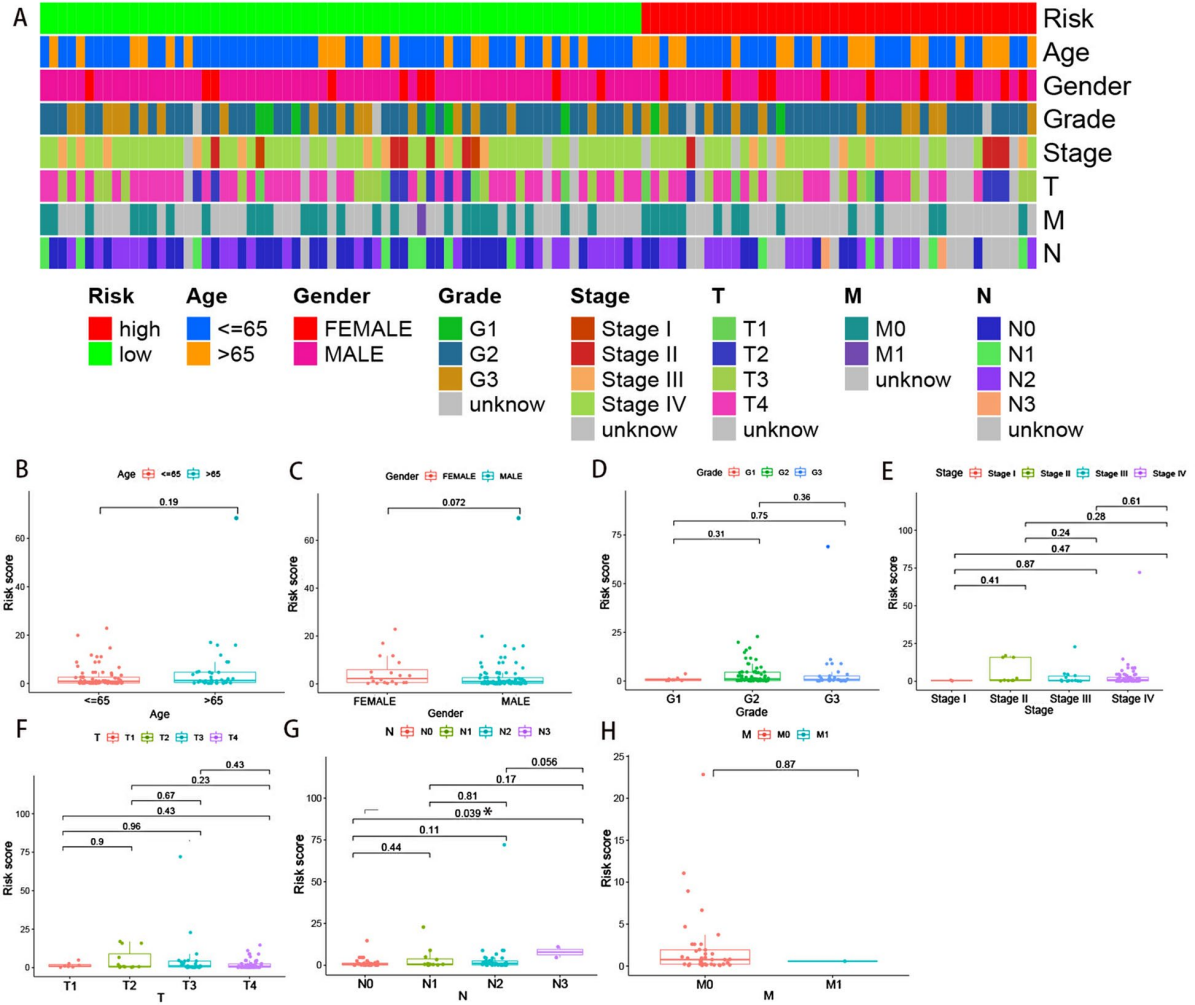
Correlations between the riskscore and other clinical characteristics. A strip chart (A) and the scatter diagrams showed the correlations between riskscore and age (B), gender (C), grade (D), stage (E), T(F), N(G), M(H)

**Fig. 6:**
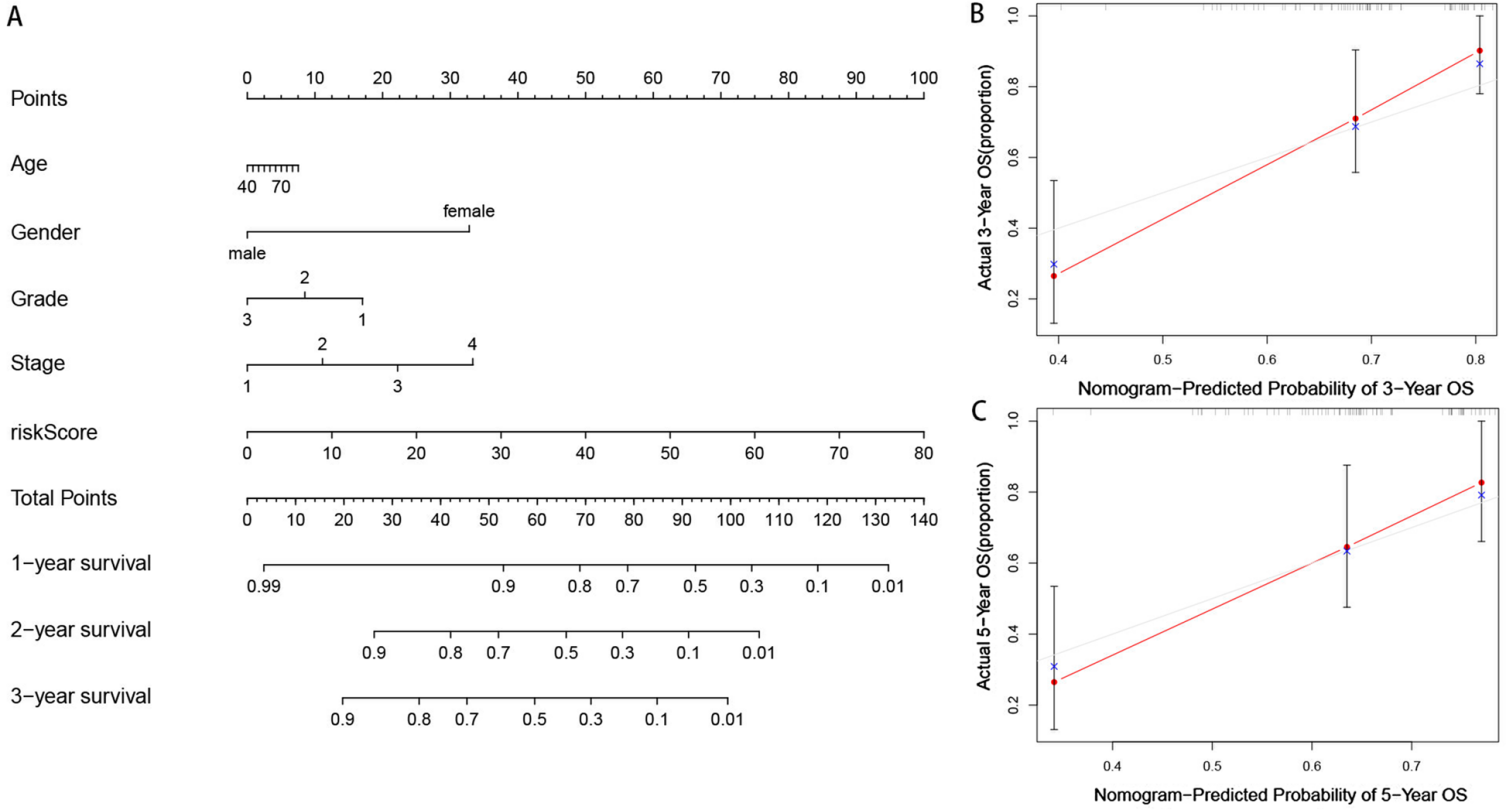
A nomogram was developed to predict survival probability. **A** A nomogram was developed in which age, gender, grade, stage and riskscore were integrated. **B** The calibration curve of 3-year survival. **C** The calibration curve of 5-year survival

Based on the 5-year riskscore ROC curve, the cutoff value was set as 1.366 (Figure 3B). Patients were then classified into the high-risk group (>1.366) and the low-risk group (<1.366). The survival rate was compared between the two groups and the result showed that the low-risk group had a better prognosis (*P*<0.001) (Figure 3C, D, E). Univariate and multivariate Cox analyses confirmed that the riskscore was an independent prognostic factor of LSCC (Figure 4A, B). Moreover, the results from the ROC curves revealed that compared with other clinical characteristics, the riskscore was better in prognostic prediction in the 1, 3, and 5 years and the area under the curves (AUCs) were 0.796, 0.946, and 0.895, respectively (Figure 4C, D, E). We also compared the differences in the riskscore between other clinical characteristics (Figure 5A-H). Our results demonstrated that N3 patients had a higher riskscore than N0 patients. To accurately predict survival probability in LSCC patients, we developed a nomograph combining age, gender, grade, stage, and riskscore (Figure 6). In the nomograph, a higher point represented a poor prognosis.

### Correlation analysis of tumor-infiltrating immune cells and riskscore values

**Fig. 7:**
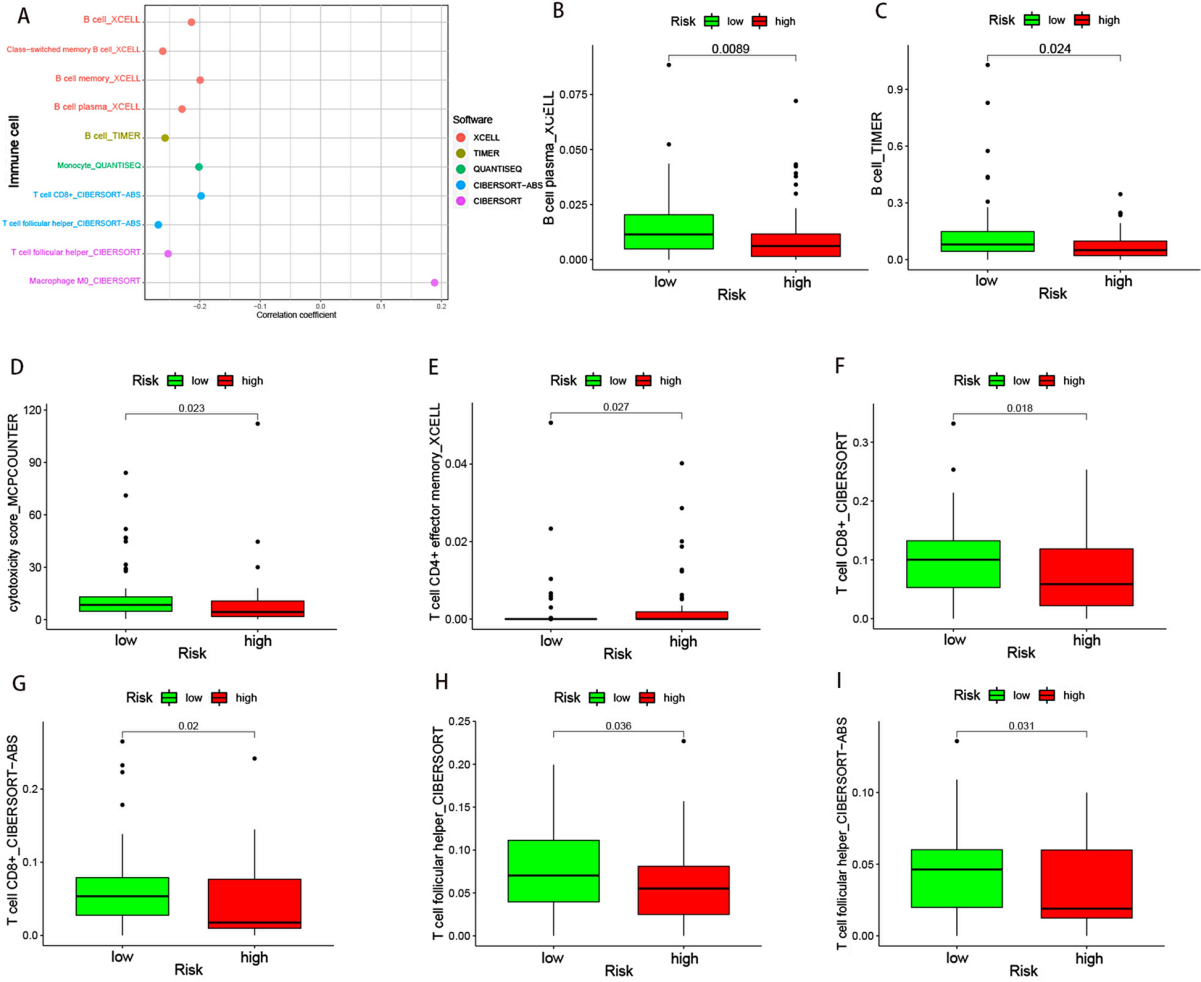
Exploration the relationship between tumor-infiltration immune cells and riskscore. (A) tumor-infiltration cells, such as B cell plasma, B cell, cytotoxicity score, T cell CD4 + effector memory, T cell CD8 + , T cell follicular helper, were negatively associated with the riskscore. The scatter diagrams showed that B cell plasma (B), B cell (C), cytotoxicity score (D), T cell CD4 + effector memory (E), T cell CD8 + (F, G), T cell follicular helper (H, I) were higher in low-risk group

**Fig. 8:**
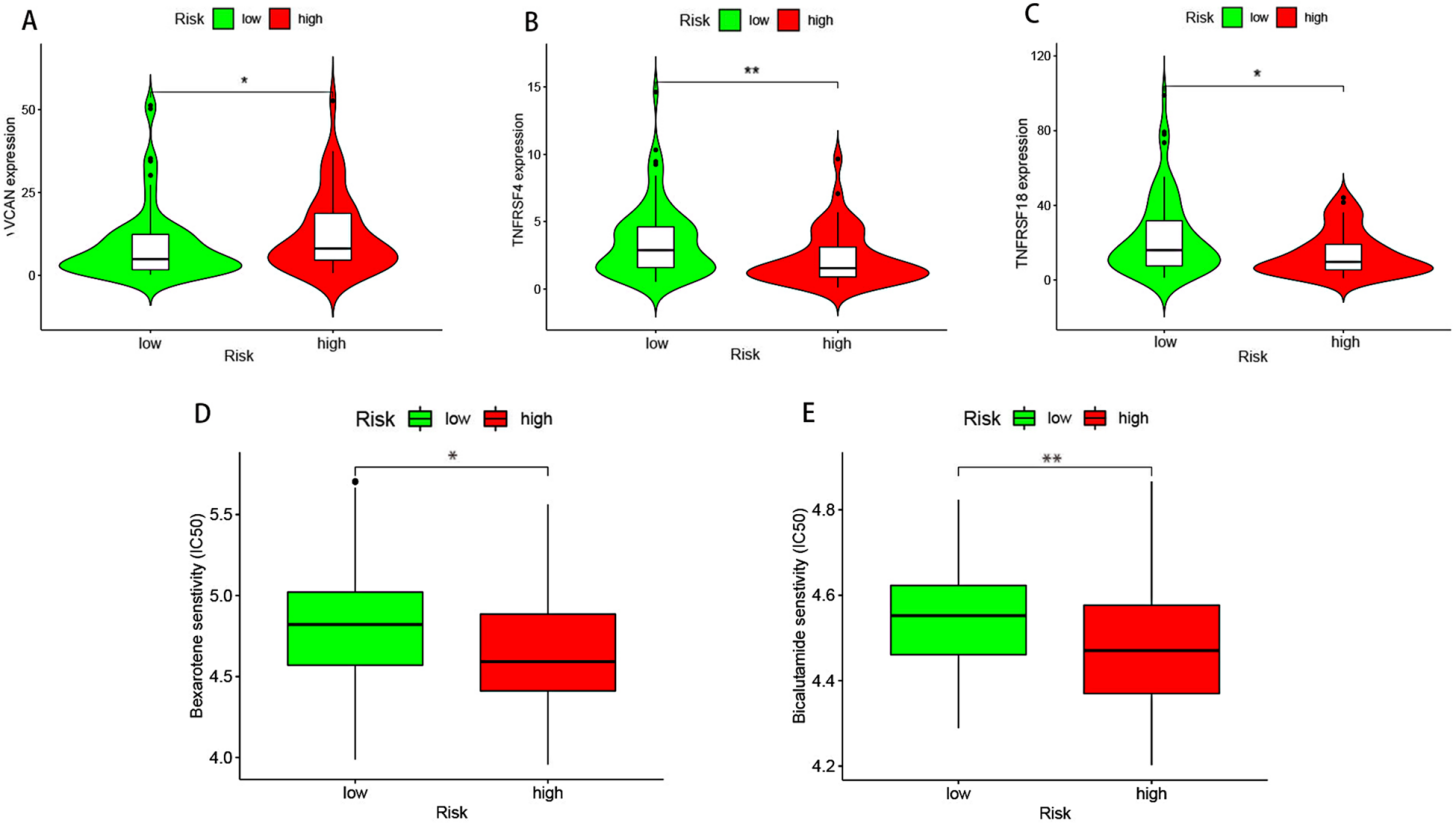
Immune related genes were differently expressed between high-and low risk groups and riskscore was a potential predictor for chemotherapeutics.VCAN (A) was highly expressed in high-risk group while TNFRSF4 (B) and TNFRSF18 (C) were highly expressed in low-risk group. Bexarotene (D) and bicalutamide (E) had lower IC50 value in high-risk group

Combining with the downloaded results from the TIMER2.0 database, we studied the correlation between the riskscore and the content of tumor-infiltration immune cells (Fig. 7A-I). We found that B cell plasma, B cell, cytotoxicity score, T cell CD4^+^ effector memory, T cell CD8^+^, and T cell follicular helper were higher in the low-risk group. We also assessed the expression of immune-related genes in different groups. TNFRSF4 and TNFRSF18 were highly expressed in the low-risk group while versican (VCAN) was highly expressed in the high-risk group (Fig. 8A-C).

### Association between the risk model and chemotherapeutics

We also investigated the correlation between risk and efficacy of common chemotherapeutics in the TCGA LSCC dataset. The results indicated that a high riskscore was associated with a lower IC_50_ of chemotherapeutics such as bexarotene and bicalutamide, suggesting that the model served as a potential predictor for chemosensitivity (Figure 8D, E).

## Discussion

Based on LSCC datasets from TCGA, we selected differently expressed immune-related lncRNAs, matched lncRNA pairs, and then successfully established a signature of immune-related lncRNA pairs. Using this signature, we scored each sample and drew 1-, 3-, and 5- year riskscore ROC curves, respectively. Subsequently, we divided patients into high- and low-risk groups according to the cutoff value of 5- year riskscore ROC curve. Riskscore was confirmed to be an independent prognostic factor of LSCC. Moreover, our result provided an accurate nomograph for predicting survival probability in LSCC patients.

LSCC is one of the most common subtypes of head and neck squamous cell carcinoma (HNSCC). Over the past decades, the mortality of LSCC has significantly increased worldwide; therefore, early detection and precise treatment are crucial [17]. Significant effort has been focused on molecular pathogenesis, development, and recurrence of LSCC. Numerous studies have proven that lncRNAs could serve as potential oncogenes or tumor suppressors in tumorigenesis and tumor progression of several types of cancers [18]. For example, the expression of lncRNA retinoblastoma-associated transcript-1 (RBAT1) was significantly higher in both retinoblastoma (Rb) and bladder cancer (BCa) clinical tissues compared with normal tissues and RBAT promoted tumorigenesis by activating E2F3 transcription [19]. In lung cancer, lncRNA JPX was reported to upregulate Twist1 by competitively sponging miR-33a-5p, subsequently inducing EMT and lung cancer cell invasion [7]. Specifically, overexpression of lncRNA MIR31HG was correlated with aggressive clinicopathological features and could serve as a poor prognostic biomarker in LSCC [20]. While LINC‐PINT expression was downregulated in laryngeal carcinoma tissues, it acted as a suppressor through the LINC-PINT/miR-425-5p/PTCH1 axis [9]. Because tumor heterogeneity limits therapeutic efficacy, using a single prognostic biomarker in LSCC patients could be unreliable [21]. Therefore, the use of multiple biomarkers has been suggested in the predictive prognosis of laryngeal cancer and the development of new therapeutic strategies. Gong et al. successfully established a 6-lncRNA model, which had good performance for predicting survival in LSCC patients to aid in risk stratification and provide precise therapeutic recommendations [22]. Zhang et al. identified a 4-lncRNA signature predicting the prognosis of patients with laryngeal cancer [23]. They reported that the signature could influence the prognosis of laryngeal cancer by regulating tumor apoptosis, metastasis, invasion, immunity, and other characteristics through the Notch and Wnt signaling pathway and voltage-gated calcium channels. In the present study, we successfully constructed a signature of 7-lncRNA pairs. To reduce the differences in the expression levels of the same lncRNAs from different samples, we adopted an lncRNA-pairing method where only the expression levels of two lncRNAs in the same sample are compared instead of comparing these lncRNAs in different samples. In addition, two pairs of lncRNAs were selected for qRT-PCR verification of their expression in LSCC, and the results were consistent with our matching principle. Practically, our signature had better prognostic prediction performance compared with other clinical characteristics such as stage, age, gender, and grade.

Malignant tumors are mainly composed of cancerous cells and are influenced by a complex microenvironment. The contexture of immune cells in the tumor microenvironment (TME) profoundly influences tumor progression and the effectiveness of anti-cancer therapies [24]. Although HNSCC modalities such as surgery, radiotherapy, and chemotherapy have significantly improved over the past decades, recurrence rates are still high [25]. Given that HNSCCs are tumors with a rich immune infiltrate, HNSCC treatment is geared towards immunotherapy [25]. Association between TME and immune infiltration in LSCC has been previously investigated [26–30]. To explore the association between riskscores and tumor-infiltrating immune cells, we used common acceptable methods, including TIMER, CIBERSORT, QUANTISEQ, xCELL, MCP-counter, and EPIC, to estimate immune-infiltrating cells. After analysis, our results demonstrated that B cell plasma, B cell, cytotoxicity score, T cell CD4^+^ effector memory, T cell CD8^+^, and T cell follicular helper were higher in the low-risk group. This suggested that more immune-infiltrating cells were associated with a better prognosis, which was in accord with previous results. Zhou et al. [28] reported higher densities of CD8^+^ cell infiltration in early tumor stages than in late tumor stages in LSCC. They also found that a high density of CD8^+^ immune cells in both peritumoral and intratumoral regions was significantly associated with a favorable OS. In our study, we assessed the expression of immune-related genes in the high- and low-risk groups, and our results indicated that TNFRSF4 and TNFRSF18 were highly expressed in the low-risk group while VCAN was highly expressed in the high-risk group. In line with our result, it was reported that gastric cancer patients with high VCAN expression had worse prognoses than those with low VCAN expression and VCAN was an independent risk factor for OS [31]. Contrary to our result, Gu et al. [32] demonstrated that a higher TNFRSF4 expression level contributed to the poor prognosis of patients with non-M3 acute myeloid leukemia (AML). This discrepancy in results could be due to different disease types or sample sizes. Since chemotherapy is one of the important treatments in LSCC, we compared the IC_50_ of different chemotherapeutic drugs in high- and low-risk groups. We found that a high riskscore was associated with a lower IC_50_ of bexarotene and bicalutamide, suggesting that our model served as a potential predictor for chemosensitivity.

With the advancement of gene sequencing techniques coupled with traditional single biomarker analyses, signatures composed of multiple genes, miRNAs, or lncRNAs have attracted more attention and may offer practical solutions by providing treatment suggestions and predicting prognosis. Zhang et al. [33] established a five-gene signature that could effectively predict the prognosis of laryngeal cancer. Cui et al. [34] explored a genome-wide integrated analysis of methylation and the transcriptome to construct a methylation-driven gene-based prognostic model to precisely predict recurrence probability and optimize therapeutic strategies for LSCC.

Meanwhile, a novel 4-lncRNA signature was identified in patients with laryngeal cancer [35]. In our study, we combined the immune-related mRNA and lncRNA expression level to successfully construct a signature of immune-related lncRNA pairs and explored its clinical significance. To the best of our knowledge, this is the first report to investigate a signature of immune-related lncRNA pairs in LSCC. We selected 7 lncRNA pairs: AL133330.1|AC132872.3, LINC01094|LINC02154, LINC02575|AC122685.1, MIR9-3HG|LINC01748, AC092687.3|SNHG12, AC090204.1|AL158166.1, and LINC01063|Z82243.1. Among these lncRNAs, some have been reported to play essential roles in different types of tumors. For example, LINC01094 was found to upregulate SLC2A3 by targeting microRNA-184, subsequently promoting the development of clear cell renal cell carcinoma [36]. SNHG12 is a potential biomarker in various cancer types, including gastric cancer, non-small cell lung cancer, triple-negative breast cancer, hepatocellular carcinoma, etc. Therefore, targeting SNHG12 could lead to advances in the diagnoses, prognosis, and/or treatment of these cancers [37].

However, this study has some shortcomings. Firstly, our results were based on the TCGA LSCC data set, which was relatively insufficient. Secondly, although we confirmed our signature in the downloaded data, some lncRNAs in the signature have not been published before, limiting validation by other databases. Therefore, our signature should be verified with large sample sizes and basic experiments.

## Conclusion

In conclusion, we successfully established a signature of immune-related lncRNA pairs that is more stable by neglecting the differences of samples. Our signature has demonstrated a better prognostic ability for LSCC patients and may assist clinicians to precisely prescribe chemo drugs. Certainly, there are also several limitations in our results. Our current datas are all downloaded from public databases, and we have not carried out comprehensive experimental verification.

## Supplementary Information

**Additional file 1.** (TIF 55 kb)

**Additional file 2.** A total of 1710 immune-related mRNAs were identified.

**Additional file 3.** A total of 1105 immune-related lncRNAs were identified by co-expression analysis.

**Additional file 4.** A total of 301 differently expressed immune-related lncRNAs were identified.

**Additional file 5.** A total of 35225 lncRNA pairs were constructed.

## Data Availability

The datasets of this study are available on request to the corresponding author.

## Abbreviations

LSCC: Laryngeal Squamous Cell Carcinoma
lncRNA: Long non-coding RNA
TCGA: The Cancer Genome Atlas
FDR: False Discovery Rate
OS: Overall Survival
ROC: Receiver Operating Characteristic
AUC: Area Under Curve
HNSCC: Head and Neck Squamous Cell Carcinoma
CRC: Colorectal cancer
TIMER: Tumor Immune Estimation Resource
VCAN: Versican
TNFRSF: Tumor Necrosis Factor Receptor Super Family
Rb: Retinoblastoma
BCa: Bladder cancer
E2F3: E2F Transcription Factor 3
EMT: Epithelial-Mesenchymal Transition
MIR31HG: MIR31 Host Gene
PTCH1: Patched 1
TME: Tumor micro-environment
AML: Acute Myeloid Leukemia
SLC2A3: Solute Carrier Family 2 Member 3
SNHG: Small Nucleolar RNA Host Gene
