# Supplementary figures and images for: An immune-related lncRNA pairs signature to identify the prognosis and predict the immune landscape of laryngeal squamous cell carcinoma
Source: BMC Cancer. 2022 May 14;22:545. doi: 10.1186/s12885-022-09524-1 (PMC9107277; doi:10.1186/s12885-022-09524-1)

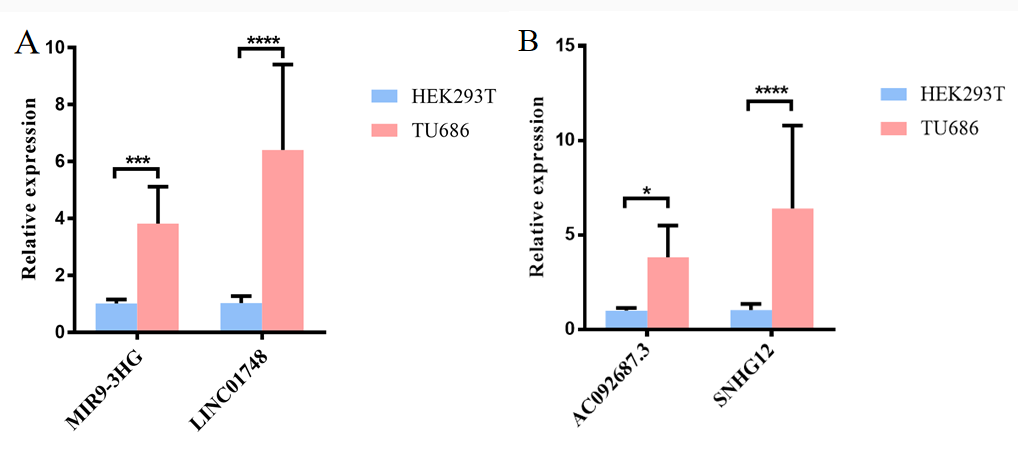

Supplement: Supplementary file 1 — Additional file 1. (TIF 55 kb) [file 12885_2022_9524_MOESM1_ESM.tif]
